# Verbal Memory Performance in Depressed Children and Adolescents: Associations with EPA but Not DHA and Depression Severity

**DOI:** 10.3390/nu12123630

**Published:** 2020-11-25

**Authors:** Sophie Emery, Isabelle Häberling, Gregor Berger, Noemi Baumgartner, Michael Strumberger, Mona Albermann, Kristin Nalani, Klaus Schmeck, Suzanne Erb, Silke Bachmann, Lars Wöckel, Ulrich Müller-Knapp, Brigitte Contin-Waldvogel, Bruno Rhiner, Susanne Walitza, Martin Hersberger, Renate Drechsler

**Affiliations:** 1 Department of Child and Adolescent Psychiatry and Psychotherapy, Psychiatric Hospital, University of Zurich, 8032 Zurich, Switzerland; isabelle.haeberling@kjpd.uzh.ch (I.H.); gregor.berger@pukzh.ch (G.B.); noemi.baumgartner@kjpd.uzh.ch (N.B.); mona.albermann@uzh.ch (M.A.); susanne.walitza@pukzh.ch (S.W.); renate.drechsler@kjpd.uzh.ch (R.D.); 2 Research Department of Child and Adolescent Psychiatry, Psychiatric University Hospitals Basel, University of Basel, 4002 Basel, Switzerland; Michael.Strumberger@upk.ch (M.S.); Klaus.Schmeck@upk.ch (K.S.); Lars.Woeckel@clienia.ch (L.W.); 3 Clinic for Psychosomatic Medicine and Psychiatry, University Hospital Zurich, 8091 Zurich, Switzerland; kristin.nalani@kjpd.uzh.ch; 4 Child and Adolescent Psychiatric Services St. Gallen, 9004 St. Gallen, Switzerland; Suzanne.Erb@kjpd-sg.ch; 5 Department of Psychiatry, University Hospitals of Geneva, 1226 Thônex, Switzerland; silke.bachmann@gmail.com; 6 Department of Psychiatry Psychotherapy, Psychosomatic Medicine, Medical Faculty of Martin Luther University, 06112 Halle, Germany; 7 Clienia Littenheid AG, 9573 Littenheid, Switzerland; 8 Child and Adolescent Psychiatry Klinik Sonnenhof, 9608 Ganterschwil, Switzerland; Ulrich.mueller-knapp@kjpz.ch; 9 Child and Adolescent Psychiatric Services Baselland, 4410 Liestal, Switzerland; Brigitte.Contin@pbl.ch; 10 Child and Adolescent Psychiatric Services Thurgau, 8570 Weinfelden, Switzerland; bruno.rhiner@stgag.ch; 11 Neuroscience Center Zurich, University of Zurich and ETH Zurich, 8057 Zurich, Switzerland; 12 Center for Integrative Human Physiology Zurich, University of Zurich, 8057 Zurich, Switzerland; Martin.Hersberger@kispi.uzh.ch; 13 Division of Clinical Chemistry and Biochemistry, University Children’s Hospital Zurich, 8032 Zurich, Switzerland

**Keywords:** omega-3 polyunsaturated fatty acid, EPA, DHA, depression, depression severity, verbal memory, children, adolescents, cognition

## Abstract

Omega-3 polyunsaturated fatty acids (n-3 PUFAs) have been described as positively associated with cognitive functioning. Current meta-analyses have identified eicosapentaenoic acid (EPA) as potentially more effective than docosahexaenoic acid (DHA). An especially vulnerable subgroup that might benefit from these beneficial effects are depressed youths. In this study, we examined associations between red blood cell (RBC) DHA and EPA levels and depression severity and verbal memory performance in a sample of 107 moderately (*n* = 63) and severely (*n* = 44) depressed youths. The findings showed that youths with high RBC EPA levels had steeper learning curves compared to those with moderate or low RBC EPA levels (Pillai’s Trace = 0.195, *p* = 0.027, η_p_^2^ = 0.097). No associations between RBC DHA levels or depression severity and verbal memory performance were observed. Our results further confirm previous findings indicating a more important role of EPA compared to DHA in relation to cognitive functioning. Future research should further investigate the differential role of EPA and DHA concerning cognitive functioning in depressed youths. Evidence supporting beneficial supplementation effects could potentially establish a recommendation for a natural and easily accessible intervention for cognitive improvement or remission.

## 1. Introduction

According to the World Health Organization (WHO), approximately 4.4% of the world’s population suffer from depression [1], with a lifetime prevalence rate of approximately 10%–15% [2,3]. Although the median age of onset lies at around 25 [2], the first symptoms and onset of depression often occur as early as during childhood or adolescence [4], and earlier onset is associated with poorer health outcomes, such as more depressive episodes, more suicide attempts, and poorer functional outcomes [5]. For adolescents, the one-year prevalence rates are estimated to lie around 5% with a large range of approximately 0.2%–17% [6,7,8,9]. Depression is a very severe mental disorder and can lead to suicide, which, in 2015, was the second leading cause of death amongst 15–29-year-olds [10]. In youths, depression has been deemed the leading cause of disability-adjusted life years (DALYs) and years lived with disability (YLD) [11]. Altogether, these findings highlight the burden that this disease poses on today’s youth and the devastating associated consequences.

The Diagnostic and Statistical Manual of Mental Disorders, Fifth Edition (DSM-5) [12] defines the diagnostic criteria for major depressive disorder (MDD), which include depressed mood, fatigue, and diminished pleasure, as well as cognitive problems such as difficulties concentrating. To meet the diagnostic criteria for MDD, at least five symptoms must have been present for most of the time during the same two-week period. Symptoms must include either depressed mood or loss of interest or pleasure. In children and adolescents, key symptoms also include irritable mood.

Clinical research to date has primarily focused on the emotional symptoms of depression, while problems with cognition can equally impact everyday functioning and subjective quality of life [13,14,15]. Moreover, cognitive deficits have been associated with poorer antidepressant treatment outcome [16,17]. Cognitive impairments could be especially problematic when children and adolescents are affected. At this critical stage in life, problems with concentration and cognition could lead to long-lasting consequences concerning personal life and educational attainment [18,19]. Meta-analyses investigating cognitive deficits in depressed adults have reported moderate impairments in several cognitive domains compared to healthy controls [20,21,22,23,24,25,26]. Evidence from several studies suggests that these cognitive problems remain at least partially present even in the remitted stage [27,28,29]. Although cognitive complaints are equally part of the diagnostic criteria in youths, evidence for impaired cognitive test performance in this subgroup of depressed individuals seems much more heterogeneous. Some meta-analyses have found similar deficits to analyses with adult patients [30,31,32]. A qualitative review by Vilgis et al. [33], however, concluded that while some studies reported cognitive deficits in depressed children and adolescents, the majority did not find any impairments. Günther et al. [34] reported heterogenous results with undisturbed attention, whereas memory impairment was associated with depressive disorders.

In their review, McDermott and Ebmeier [35] reported negative associations between depression severity and cognitive functioning in adults; however, this effect seemed domain-specific. In youths, thus far only tendencies toward a similar association have been reported [36], highlighting the need for further investigation into this subject. In the past few years, cognitive remission has become an important goal for MDD treatment; however, treatment options concerning cognitive symptoms are scarce. Evidence of positive cognitive effects of antidepressant medication has proven rather heterogeneous [37,38,39,40,41,42,43,44].

Since the industrial revolution, nutritional patterns in Western societies have shifted toward foods containing more omega-6 (n-6) compared to omega-3 (n-3) polyunsaturated fatty acids (PUFAs) [45]. These changes have been held responsible for the rise in different civilization diseases such as cardiovascular diseases, but also in psychiatric diseases such as depression [46]. n-3 PUFA deficiency has been associated with depression [47,48,49] and eicosapentaenoic acid (EPA) status has been negatively associated with depression severity [50]. Meta-analytic evidence has further suggested beneficial effects of n-3 PUFA supplementation on depression symptoms [51,52,53,54,55], especially with high doses of EPA [51,52]. Interestingly, symptom severity might moderate this beneficial effect, as some studies have reported stronger effects in more severely depressed patients [56]. However, other meta-analyses in both adults [57] and children and adolescents [58] have revealed no beneficial effect of n-3 PUFAs for the treatment of depressive disorder. In youths, however, only four randomized controlled trials (RCTs) thus far have been included [58]. The anti-inflammatory properties of n-3 PUFAs have mostly been made responsible for their beneficial effects in relation to depression [59]. Proinflammatory cytokines have been shown to play a role in depression [60], and hence n-3 PUFAs have been discussed to counteract these inflammatory processes [61].

n-3 PUFAs are vital for normal brain development [61,62,63,64]. Consequently, the past few years have seen a renewed interest in the investigation of n-3 PUFAs in relation to cognition. Several studies in youths have reported positive associations between reported dietary n-3 PUFA intake and cognitive performance [65,66]. In a study by Montgomery et al. [67], low blood n-3 PUFA status was associated with poorer reading abilities and working memory performance in children. Similarly, Wurff et al. [68] reported better performance on an attention task for adolescents with higher n-3 PUFA status. Meta-analyses of n-3 PUFA supplementation have reported mixed results, depending on the study population investigated [69,70,71,72,73,74,75,76]. It remains unclear whether the specific type of n-3 PUFA ingested might have significantly contributed to these heterogenous study results, as some meta-analyses have suggested beneficial effects of EPA rather than docosahexaenoic acid (DHA) [69,77].

The question remains as to how n-3 PUFAs might affect cognition in depressed individuals, as supplementation studies in depressed populations are very scarce [78]. The results reported by Rogers et al. [79] showed no benefit of n-3 PUFA supplementation on cognition in adult individuals with mild to moderate depression. In youths, however, Vesco et al. [80] reported decreased parent-rated impairments in executive functioning after 12-week supplementation with n-3 PUFAs. To the best of our knowledge, there is as yet no study investigating the associations between EPA and DHA statuses and cognitive performance in depressed children and adolescents.

The aim of the current study was to address the previously mentioned research deficits and to investigate verbal memory performance in depressed children and adolescents in relation to EPA and DHA statuses as well as depression severity. The aforementioned research findings suggest that (1) EPA but not DHA is positively associated with cognitive functioning, (2) depression severity is negatively associated with cognitive functioning, and (3) depression severity might moderate the association between EPA and depression symptoms. We hence hypothesized that patients with a high EPA status would outperform patients with a low EPA status in verbal memory tasks and that this would be especially evident in severely depressed compared to moderately depressed individuals. Furthermore, we hypothesized that moderately depressed patients would outperform severely depressed patients.

If EPA but not DHA were to be identified as being associated with cognitive functioning in depressed children and adolescents, this would prove important, especially for this specific population, as EPA compared to DHA has also been considered to be more effective in the treatment of depression symptoms [51,52]. Consequently, future RCTs could investigate EPA supplementation as an intervention in these individuals, targeting both emotional and cognitive symptoms at the same time.

## 2. Materials and Methods

The findings reported in this present study were generated from data collected by the “The Omega-3-pMDD trial,” a multi-center placebo-controlled trial aiming to investigate the efficacy of omega-3 fatty acid supplementation in moderately to severely depressed children and adolescents aged 8–17 years. Two hundred and twenty patients across Switzerland will be recruited in order to investigate both the psychopathological and cognitive effects of nine months of n-3 PUFA supplementation. In the present study, we report cross-sectional findings from data collected prior to randomization to a treatment arm, with the exception of intellectual ability that was assessed six weeks post-randomization. Funding was received from the Swiss National Foundation and the private foundations that are listed later in the funding section. No industry funding was received. The clinical trial was registered on www.ClinicalTrials.gov, protocol no. NCT03167307. The clinical trial’s design paper has already been published [81]. The study was approved by the local ethics committee and was conducted in accordance with the ethical standards laid down in the 1964 Declaration of Helsinki and later amendments. All parents (or legal guardians) provided written informed consent and written or oral assent was obtained from the participating children before entering the study.

### 2.1. Participants

Participants were recruited from seven different in- and outpatient services across Switzerland. For inclusion, the patients had to meet the diagnostic criteria for MDD according to the Diagnostic and Statistical Manual of Mental Disorders (DSM)-IV [82] and had to have reported symptoms of at least moderate severity defined by a Children’s Depression Rating Scale [83] total score of ≥40 [84]. Patients who met the DSM-IV diagnostic criteria for an eating disorder within the last six months or the diagnostic criteria for a lifetime diagnosis of schizophrenia, bipolar affective disorder, or substance dependency were excluded from the trial. Mental retardation, pervasive development disorder, or pre-existing neurological or medical conditions that are likely to be responsible for depression symptoms constituted further exclusion criteria. Antidepressant treatment was allowed at study entry to avoid selection bias toward less severely depressed patients. Lastly, patients regularly supplementing n-3 PUFAs within the last six months (dosage limit set at 600 mg/day) or patients unable to follow the study procedures (e.g., due to a language barrier) were also excluded.

During the screening visit, the inclusion and exclusion criteria were assessed using the Kiddie Schedule for Affective Disorders and Schizophrenia (K-SADS-PL) [85] and the Children’s Depression Rating Scale revised (CDRS-R) [83]. Patients meeting all inclusion and no exclusion criteria were then included in the lead-in phase of the trial (7–14 days). During the lead-in phase, all participants received a placebo in a single blinded fashion and psychopathological, cognitive, and bloodwork baseline data were collected. Assessment of the inclusion and exclusion criteria was repeated at the end of this phase, after which patients were randomized to a treatment arm in a double-blind fashion. At the time of analysis, the sample consisted of 107 patients that met all of the inclusion and no exclusion criteria at screening and had analyzed PUFA blood sample data. The current article investigated this subsample in a cross-sectional manner using single cognitive and psychopathological assessments from the lead-in phase of the trial.

### 2.2. Instruments

#### 2.2.1. Sociodemographic Variables

Sociodemographic information and information on treatment history were obtained using the patients’ medical records, as well as patient and parent interviews. Information on the course of illness was collected via patients’ self-reports and parents’ reports. The patients’ medical records were consulted for confirmation of these reports. Patients or parents provided further information where information was missing or incomplete.

#### 2.2.2. Independent Variables

##### Severity of Depression (CDRS-R)

The CDRS-R is a semi-structured clinical interview assessing the severity of depression using 17 depression symptoms [83]. Patients and parents provide information on 14 of these symptoms which are then rated on a 7- or 5-point Likert scale, depending on the specific symptom. The interviewer then integrates this information and provides a final score for each symptom. Three further nonverbal symptoms are rated by the interviewer only (depressed facial affect, hypoactivity and listless speech). The final scores for each reported symptom, as well as the ratings on nonverbal symptoms are added up to obtain a final score for depression severity. Scores of 30–39 can be interpreted as mild, 40–59 as moderate, and ≥60 as severe symptoms of depression [84]. Validation studies on CDRS-R have reported good validity and reliability for depressed children and adolescents [86].

##### EPA and DHA Statuses

Venous blood samples were collected. For determination of erythrocyte fatty acid composition, blood was drawn into EDTA tubes, centrifuged, and then the plasma and buffy coat taken off; the erythrocytes were then frozen at −80 °C until analysis. The fatty acid composition of the erythrocytes was analyzed by gas chromatography at Omegametrix GmbH using previously described methods [87]. EPA and DHA statuses are given as a percentage of total fatty acids measured in RBCs.

#### 2.2.3. Outcome Variables

##### Cognitive Tests—Memory

- Verbal Memory: VLMT
  We used a validated German version (Verbaler Lern- und Merkfähigkeitstest (VLMT) [88]) of the Auditory Verbal Learning Test (AVLT) [89], in which a list of 15 semantically independent words is presented auditorily to an individual and he or she is asked to remember and reproduce as many words as possible. This process is repeated five times. Then, a second list of 15 words (= interference list (I)) is presented and the individual is asked to remember and reproduce as many words as possible from the second list. In the next step, the individual is asked to reproduce the words from the first list. After 20–30 min, he or she is once again asked to reproduce the first list of words. In the last step, the individual is asked to recognize the words from a list of 50 semantically or phonetically related and unrelated words. The test measures declarative verbal memory capacity. Short-term verbal memory is characterized by the number of words correctly reproduced by the individual in each of the five rounds (T1, T2, T3, T4, and T5). The long-term memory parameters are T7, which is the number of words from the first list recalled after 20–30 min, and T5–T7, which is the difference between the number of words recalled at T5 and T7. The interference score is I, which represents the number of correctly reproduced words from the interference list, T6, which is the number of words from the first list recalled after interference, and T5–6, which is the difference between the number of words reproduced from the first list before and after interference. Lastly, recognition (W) is the number of words correctly identified as belonging to the first list, and W–F is the correctly identified words minus the words wrongly attributed to the first list.
- Numeric Memory: WISC-IV Digit Span
  The digit span subtest from the Wechsler Intelligence Scale for Children—Fourth Edition (WISC-IV) [90] consists of two parts, namely, forward and backward. In the first part, the individual has to reproduce sequences of digits of increasing length. In the second part, the individual is instructed to repeat a sequence of digits in reverse order. The test measures numeric short-term memory and working memory.

#### 2.2.4. Control Variables

##### IQ: Reynolds Intellectual Assessment Scales and Screening (RIAS)

The RIAS is an intelligence test for individuals between 3 and 99 years of age, mostly used for research purposes [91]. It includes two subtests, the results of which are added to form a verbal intelligence index (VIX) and two subtests to form the nonverbal intelligence index (NIX). These are then integrated into the global intelligence index (GIX). Reliability of the RIAS is high (Cronbach α = 0.95 for the global index) [92,93].

##### C-Reactive Protein (CRP)

High-sensitive CRP was measured in the plasma in an accredited medical laboratory using a commercial assay with a coefficient of variation of 1.3% at 3.6 mg/L, to be used as a marker for low-grade inflammation and as a stratification parameter for randomization. Values above 10 were excluded from analysis, as they could have indicated infection.

##### Body Mass Index (BMI)

The BMI was calculated using weight (kg)/height (cm)^2^. BMI was not obtained in some cases because of missing measurement tools and devices.

### 2.3. Statistical Analysis

Statistical Package for the Social Sciences (SPSS) software version 26 was used for all statistical analyses. Descriptive statistics are given in order to describe the study sample characteristics. Moderately and severely depressed patient groups were distinguished using a clinical cut-off of the CDRS-R total score (40–59 = moderate; ≥60 = severe) [84]. Three approximately equally sized groups were formed according to the data distribution of EPA and DHA statuses, because the patients’ n-3 PUFA statuses were all low when referring to the currently proposed ideal levels [94]. Hence, the formation of three groups would allow for the investigation of especially low status and rather normal values. Differences between moderately and severely depressed patients and differences between EPA and DHA status groups for the investigation of potential confounding variables were analyzed using chi-square tests, independent *t*-tests, and one-way ANOVAs, depending on the scale level of the variable investigated. Non-parametric Mann–Whitney *U* or Kruskal–Wallis tests were only used for strongly skewed data (>1, <(−1)), as in large sample sizes parametric tests should be robust against the violation of normality assumption [95].

In order to investigate differences in memory scores depending on depression severity (moderate vs. severe) and EPA and DHA statuses (low, intermediate, and high), multivariate analyses of covariance (MANCOVAs) were computed to evaluate the VLMT short-term, long-term, and interference parameters separately. Multivariate outliers were checked using Mahalanobis’ distances. Bonferroni correction was applied for the interpretation of between-subjects effects and pairwise comparisons. For digit span parameters, univariate analyses of covariance (ANCOVAs) were used for the forward and backward parameters separately.

Conservative results using Pillai’s Trace instead of Wilk’s Lambda are reported [96]. Pillai’s Trace values range from 0 to 1 with increasing values indicating a stronger contribution of the independent variable to the model. For all MANCOVAs, normal distribution within groups was investigated using Shapiro–Wilk tests and Q-Q plots where *n* ≤ 25. Box’s tests were used to evaluate the homogeneity of variance–covariance matrices. Levene’s tests were used to assess the equality of error variances, although the results should be robust against violation if none of the standard deviations are more than four times as large as the corresponding smallest standard deviation [97]. The homogeneity of the regression slopes was assessed for all covariates. Significance levels were set at *p* < 0.05.

## 3. Results

At the time of analysis, the subsample of patients that met all inclusion and no exclusion criteria and where blood samples were available consisted of 107 patients. For one patient, the WISC digit span scores were missing due to the fact that the test had not been done with this individual. RIAS scores for seven patients were missing, because IQ testing was done six weeks post-randomization and these patients had not yet reached week six of the study.

### 3.1. Descriptive Statistics

Patients achieved average mean normative scores for both the VLMT total learning parameter (∑T-15) (T-score, *M* = 49.15, *SD* = 9.21), as well as the WISC digit span score (scaled score metric, *M* = 9.57, *SD* = 2.43). However, a significant proportion of patients achieved below average scores, with 24 patients (22.42%) with T-scores < 40 for the VLMT; moreover, 24 patients (22.64%) achieved scaled metric scores <8 for the digit span test. There was one outlier for the long-term memory scores, with a Mahalanobis’ distance of 25.72 (critical value = 13.82); however, it was decided not to exclude this participant because all other memory parameters were within normal range. Only 5.8% achieved recognition normative scores below average and 67% recognized all 15 words from the list. The skewness of the recognition data was −2.32 (*SE* = 0.23) and kurtosis was 6.51 *(SE* = 0.47). A total of 46.2% of the participants reached maximum recognition scores even when subtracting interference or false positive mistakes (skewness of −1.691 (*SE* = 0.25) and kurtosis of 2.96 (*SE* = 0.47)). Hence, it was decided to investigate depression severity and EPA/DHA group differences concerning recognition scores using nonparametric Kruskal–Wallis and Mann–Whitney *U* tests instead of MANCOVAs.

Severity group formation resulted in a group of 63 moderately depressed patients and a group of 44 severely depressed patients. The sociodemographic information, IQ, physiological parameters, and psychopathology data are summarized in Table 1 for both severity groups. Severely depressed patients were over proportionally female compared to moderately depressed patients; hence, gender was later entered as a covariate for the ANCOVA and MANCOVA analyses. No other group differences except CDRS-R score differences presented between severity groups. Importantly, no differences concerning antidepressant use were found.

EPA group formation resulted in three groups (*n*_1_ = 34, *n*_2_ = 39, and *n*_3_ = 34) with group means of *M*_1_ = 0.34 (*SD* = 0.05), *M*_2_ = 0.46 (*SD* = 0.04), *M*_3_ = 0.66 (*SD* = 0.11). No gender (χ^2^ = 0.172, *p* = 0.918), age (*F*(2,104) = 0.816, *p* = 0.445), IQ (*F*(2,97) = 2.317, *p* = 0.104), BMI (*H*(2) = 2.950, *p* = 0.229), CRP (*H*(2) = 2.771, *p* = 0.250) differences or differences concerning antidepressant use (χ^2^ = 0.866, *p* = 0.649) presented between the three groups. DHA group formation resulted in three groups (*n*_1_ = 36, *n*_2_ = 35, and *n*_3_ = 36) with group means of *M*_1_ = 2.89 (*SD* = 0.22), *M*_2_ = 3.48 (*SD* = 0.20), and *M*_3_ = 4.54 (*SD* = 0.61). Moreover, between these groups, no gender (χ^2^ = 0.468, *p* = 0.791), age (*F*(2,104) = 0.774, *p* = 0.464), IQ (*F*(2,97) = 0.924, *p* = 0.401), (*H*(2) = 0.987, *p* = 0.611), CRP (*H*(2)= 0.539, *p* = 0.764) differences or differences concerning antidepressant use (χ^2^ = 0.527, *p* = 0.768) were found.

As shown in Table 2, severely depressed patients had higher DHA and total omega-3 status compared to moderately depressed patients. No significant differences concerning EPA status were observed.

### 3.2. Main Analysis—EPA Status and Depression Severity in Relation to Memory

**Figure 1:**
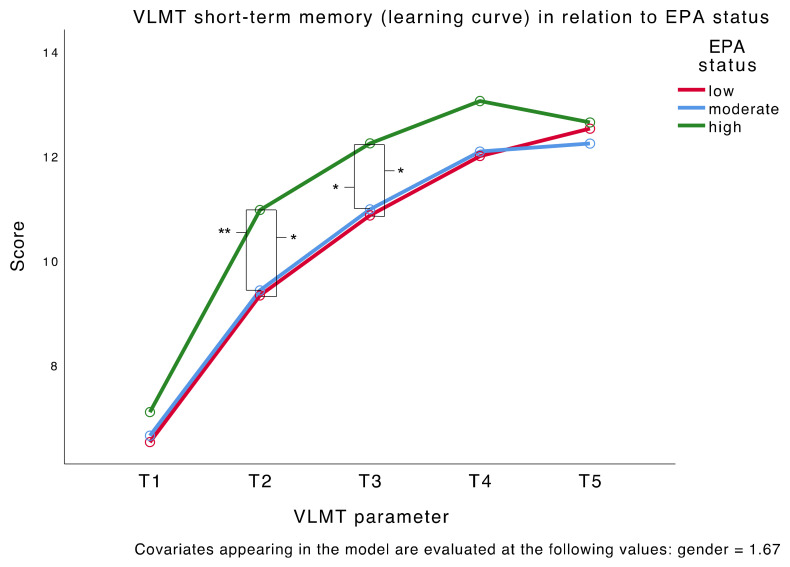
VLMT learning curve in relation to EPA status. *N* = 107. * *p* < 0.05, ** *p* < 0.01.

The MANCOVA results for the short-term memory VLMT parameters resulted in a significant main effect for EPA status (*F*(10,194) = 2.094, *p* = 0.027) but not for depression severity (*F*(5,96) = 0.622, *p* = 0.684). The between-subjects effects of EPA status were significant for the second trial (T2) (*F*(2,100) = 6.096, *p* = 0.003) and reached borderline significance for T3 (*F*(2,100) = 4.825, *p* = 0.010) after Bonferroni correction of the significance level (*p* < 0.01). Pairwise comparisons showed that patients with a high EPA status (*M* = 10.96, *SE* = 0.37) outperformed patients with a moderate (*M* = 9.42, *SE* = 0.35, *p* = 0.009) or low (*M* = 9.32, *SE* = 0.40, *p* = 0.010) EPA status in T2. The same results presented for T3, where patients with a high EPA status (*M* = 12.23, *SE* = 0.35) outperformed patients with a moderate (*M* = 10.97, *SE* = 0.33, *p* = 0.027) or low (*M* = 10.86, *SE* = 0.37, *p* = 0.023) EPA status. The VLMT short-term memory score profiles for the three EPA status groups are shown in Figure 1. The results indicate that patients with a high EPA status had steeper learning curves compared to those with a moderate or low EPA status. No main effect of either EPA status or depression severity presented for interference, long-term memory parameters, and the parameters of the digit span test. All results are summarized in Table 3. Independent samples Kruskal–Wall tests showed no effect of EPA status for either the uncorrected (*H*(2) = 0.893, *p* = 0.640) or the corrected (*H*(2) = 2.239, *p* = 0.326) recognition parameter; there was also no significant effect of depression severity (*U* = 1370.500, *p* = 0.901 *U* = 1274.000, *p* = 0.580). Further analyses concerning potential confounding group differences revealed no significant IQ differences between the six EPA/severity groups.

### 3.3. Main Analysis—DHA Status and Depression Severity in Relation to Memory

The same analyses were run using DHA instead of EPA status as a factor. A significant interaction effect for long-term memory parameters was found (*F*(4,200) = 3.069, *p* = 0.018). However, in contrast to the EPA analyses where no IQ differences were found between the groups, further analyses revealed significant IQ differences between moderately and severely depressed patients in the moderate DHA status group. Moderately depressed patients had significantly higher GIX scores (*M* = 106.05, *SD* = 8.56) compared to severely depressed patients in the moderate DHA status group (*M* = 97.62, *SD* = 10.57; *t*(31) = 2.537, *p* = 0.016). Hence, the MANCOVA and ANCOVA analyses were rerun entering IQ as a second covariate. A significant main effect for DHA status was found for digits forward scores (*F*(2,91) = 3.342, *p* = 0.040). However, pairwise comparisons were no longer significant after Bonferroni correction for multiple comparisons. No significant main effect for depression severity presented for any memory parameter. A borderline significant interaction effect presented for interference parameters of the VLMT (*F*(6,182) = 2.219, *p* = 0.052). Between-subjects effects revealed a significant effect for the T6 parameter (*F*(2,92) = 4.337, *p* = 0.016) where severely depressed patients with a high DHA status (*M* = 12.56, *SE* = 0.50) outperformed severely depressed patients with a moderate DHA status (*M* = 10.50, *SE* = 0.61, *p* = 0.036). Moreover, in the moderate DHA status group, moderately depressed patients (*M* = 12.34, *SE* 0.47) outperformed severely depressed patients (*M* = 10.50, *SE* = 0.61, *p* = 0.021). IQ proved a significant covariate for nearly all parameters. All MANCOVA and ANCOVA results are summarized in Table 4. Independent samples Kruskal–Wallis tests showed no effect of DHA status for either the uncorrected (*H*(2) = 0.522, *p* = 0.770) or the corrected (*H*(2) = 2.054, *p* = 0.358) recognition parameter. 

## 4. Discussion

The current study investigated RBC EPA and DHA levels, as well as depression severity in relation to memory performance in a sample of 107 moderately to severely depressed youths.

Contrary to previous findings that reported inverse associations between oily fish intake and depression severity [98], in our sample severely depressed patients had significantly higher DHA and total omega-3 statuses compared to moderately depressed patients. Whereas negative associations between EPA status and depression severity have been reported [50], we found no significant differences concerning EPA status between moderately and depressed patients.

In our study, we examined potential cognitive deficits in depressed children and adolescents that have been inconsistently found in previous studies [30,31,32,33,34] in contrast to research conducted in adult patient groups, where deficits seem more consistent [20,21,22,23,24,25,26]. Although most patients achieved average normative scores for both the VLMT and the digit span test, over 20% of the patients (compared to an expected 15.9% in a healthy population) achieved below average normative scores. These results indicate a tendency toward impaired verbal memory in depressed youths.

We further aimed at investigating potential negative associations between depression severity and cognitive functioning in depressed youths that so far have mostly been reported in adult patient groups [35,99], with similar tendencies from research conducted in children and adolescents [36]. Contrary to our hypothesis, in our sample we did not find any significant differences in verbal memory performance between moderately and severely depressed patients. Hence, we could not confirm any negative associations between depression severity and cognitive functions previously reported in adult patient groups [35,99].

Most importantly, we were interested in memory performance in relation to EPA and DHA status, because EPA supplementation has mostly been associated with beneficial cognitive effects in youths [69,77], and beneficial cognitive effects would prove especially important in this vulnerable population. Our results indicated that patients with a high EPA status had steeper learning curves across the short-term memory trials of a verbal list learning test, compared to patients with a moderate or low EPA status. Compared to patients with a moderate or low EPA status, those with a high EPA status, seemed to remember more words faster, although they achieved equal scores in the first trial and again in the last two trials. This finding only partly confirmed our hypothesis that patients with a high EPA status would outperform patients with a low EPA status in verbal memory tests, because differences were only observed for the second and third trials and not overall. Moreover, these differences were only found for short-term memory parameters, whereas no significant effect for EPA status presented for any other VLMT parameter or the digit span test. However, we were able to confirm our hypothesis concerning DHA by finding no significant effect of DHA on any verbal memory parameter. Both results are in line with previous findings from meta-analyses on supplementation effects of EPA and DHA that suggested beneficial effects of EPA but not DHA on cognitive functioning [69,77] and effects in clinical rather than healthy populations [69]. In contrast to our hypothesis, we were not able to confirm any superior effects of a high EPA status in severely compared to moderately depressed patients.

A number of limitations should be kept in mind when interpreting the reported results. First, no healthy control group was investigated; hence, no comparison between depressed and healthy children and adolescents could be made. Nevertheless, our data suggested that a somewhat larger proportion of participants than expected in a healthy population achieved below average normative scores in both tests. Importantly, the current study was based on a cross-sectional design. It is hence limited by the impossibility to draw conclusions about any causal relationships. Another methodological limitation lies in the small and unequally sized subgroups formed by the two factors of depression severity and DHA and EPA status. Moreover, for several analyses the Levene’s tests proved significant. However, rather conservative statistical methods for correction were applied throughout the analyses, minimizing the probability of a type-I error.

Our results further contribute to previous findings that reported some impairment considering cognitive functions [30,31,32] and especially memory performance [34] in depressed youths. Future studies should investigate differences between healthy and depressed individuals concerning memory performance in order to be able to further confirm these findings and make recommendations for cognitive assessments in this population. Surprisingly, the analyses of our sample revealed higher DHA and total omega-3 status in severely compared to moderately depressed patients. One explanation for this finding might lie in altered nutritional patterns in severely compared to moderately depressed youths. Moreover, considering the population investigated, low n-3 PUFA levels were expected to begin with, and hence, small ranges were presented. Wider ranges might therefore have produced different results. This explanation is, of course, only speculative. Although the reported analyses with EPA and DHA only contributed associations between RBC levels and verbal memory rather than effects of supplementation, they further corroborate evidence from meta-analyses that confirmed the beneficial supplementation effects of EPA but not DHA. Thus, our findings suggest that an increased dietary intake of EPA might prove beneficial for cognitive improvement or even remission in depressed youths. Based on our findings, future RCTs should investigate the effects of EPA compared to DHA supplementation in depressed children and adolescents to potentially establish a recommendation for a natural and easily accessible intervention for cognitive improvement or remission in depressed youths. Neuroimaging studies have tried to explain differences in supplementation effectivity between DHA and EPA. Bauer et al. [100], for example, reported a reduction in functional activation in the left anterior cingulate cortex and shorter reaction times in the Stroop color–word task following EPA rather than DHA supplementation, which, they argued, might represent stronger neural efficiency in EPA-supplemented compared to DHA-supplemented individuals. Biologically, however, the supplementation advantages of EPA over DHA have yet to be explained, considering EPA is found in much smaller concentrations within the brain compared to DHA [61,101]. As there is also a lack of understanding about the actual absorption and utilization of both EPA and DHA, it would be paramount to investigate the moderating effects of, for example, the microbiome, as differences might be expected, and these again might explain differential effects in different populations.

## Figures and Tables

**Table 1:** Descriptive statistics for sociodemographic information, biological parameters, IQ, psychopathology.

Sample Characteristics	Variable Specifics	*n*	*M* (*SD*)	Min	Max	Moderate Depression *M (SD)*	Severe Depression *M (SD)*	*t*/*χ*^2^/*U*	*p*
**Sociodemographic information**	Age	107	15.50 (1.89)	8.67	18.00	15.25 (2.09)	15.85 (1.51)	−1.630	0.106
	Sex: %female	107	67%			57%	82%	7.166	0.007 **
**Physiological parameters**	BMI	99	22.35 (4.87)	14.00	39.80	21.86 (4.35)	23.06 (5.52)	1285.0	0.454
	CRP	105	0.79 (1.26)	0	6.6	0.65 (0.81)	0.99 (1.69)	1220.0	0.410
**IQ**									
RIAS	VIX	100	101.73 (9.93)	79	129	102.58 (10.18)	100.51(9.61)	1.019	0.102
	NIX	100	106.33 (8.93)	70	123	107.71 (8.61)	104.34 (9.12)	1.879	0.310
	GIX	100	104.52 (9.35)	76	128	105.78 (9.32)	102.68 (9.21)	1.651	0.102
**Depression severity**									
CDRS-R	Total score	107	57.73 (7.76)	42	79	52.37 (4.18)	65.41 (4.54)	−15.331	<0.001 ***
	Severe % ^a^	44	41.1%			-	-	-	-
**Course of illness**									
Mean duration of depression (months)	Months	104	14.71 (11.69)	1	84	13.45 (9.76)	16.43 (13.84)	1503.0	0.228
Total number of episodes		105	1.46 (0.95)	1	8	1.44 (1.10)	1.48 (0.70)	1490.0	0.230
Recurrent depression	Yes	104	31%			26%	38%	1.775	0.183
**Use of antidepressant medication**	Yes	102	37%			35%	40%	0.317	0.573

Note: ** *p* < 0.01, *** *p* < 0.001. CRP, C-reactive protein; BMI, body mass index; VIX, verbal intelligence index; NIX, nonverbal intelligence index; GIX, global intelligence index; RIAS, Reynolds Intellectual Assessment Scales and Screening; CDRS-R, Children’s Depression Rating Scale revised. ^a^ CDRS-R severity scores: 40–59 = moderate, ≥60 = severe.

**Table 2:** Fatty acid status for moderately and severely depressed patients.

	Total	Moderate MDD (*n* = 63)	Severe MDD (*n* = 44)	*t*	*p*
Fatty acid	*M* (*SD*)	*M* (*SD*)	*M* (*SD*)		
EPA	0.49 (0.15)	0.47 (0.14)	0.51 (0.16)	−1.515	0.133
DHA	3.63 (0.79)	3.50 (0.71)	3.82 (0.87)	−2.077	0.040 *
All n-6	34.37 (1.32)	34.45 (1.26)	34.32 (1.40)	0.512	0.609
All n-3	6.45 (1.0)	6.26 (0.93)	6.71 (1.06)	−2.318	0.022 *
AA/EPA	35.10 (11.27)	36.08 (11.21)	33.74 (11.60)	1.045	0.298

Note: * *p* < 0.05. EPA, eicosapentaenoic acid; DHA, docosahexaenoic; n-6, omega-6; n-3, omega-3; AA, arachidonic acid.

**Table 3:** Multivariate analysis of covariance (MANCOVA) and univariate analysis of covariance (ANCOVA) results for Verbaler Lern- und Merkfähigkeitstest (VLMT) and digit span parameters. EPA status and depression severity entered as factors and gender as a covariate.

	MANCOVA EPA status (EPA) Depression Severity (S) Interaction Severity * EPA Status (I) Covariate: Gender (G)							
VLMT short-term memory parameters	EPA: *F*(10,194) = 2.094, *p* = 0.027 *, η_p_^2^ = 0.097 S: *F*(5,96) = 0.622, *p* = 0.684, η_p_^2^ = 0.031 I: *F*(10,194) = 0.910, *p* = 0.525, η_p_^2^ = 0.045 G: *F*(5,96) = 1.287, *p* = 0.276, η_p_^2^ = 0.063							
	Moderate depression *N* = 63 *M (SD)*	Severe depression *N* = 44 *M (SD)*	*F* *p* η_p_^2^				Pairwise	*p*
			EPA	S	I	G		
**T1 score**								
Low EPA	6.52 (0.33)	6.55 (0.53)	1.044	0.816	0.415	0.6	NS	NS
Moderate EPA	7.00 (0.35)	6.29 (0.46)	0.356	9.369	0.661	0.44		
High EPA	7.11 (0.40)	7.06 (0.42)	0.02	0.008	0.008	0.006		
**T2 score**								
Low EPA	9.74 (0.52)	8.91 (0.69)	**6.096**	1.577	2.03	0	EPA: h > m EPA: h > l	*p* = 0.009 ** *p* = 0.010 *
Moderate EPA	10.14 (0.56)	8.71 (0.46)	0.003 *	0.212	0.137	0.997		
High EPA	10.67 (0.26)	11.25 (0.44)	**0.109**	0.016	0.039	0		
**T3 score**								
Low EPA	11.43 (0.40)	10.36 (0.64)	**4.825**	1.674	1.366	2.47	EPA: h > m EPA: h > l	*p* = 0.027 * *p* = 0.023 *
Moderate EPA	11.27 (0.52)	10.71 (0.45)	**0.010 °**	0.199	0.26	0.119		
High EPA	11.94 (0.38)	12.50 (0.52)	**0.088**	0.016	0.027	0.024		
**T4 score**								
Low EPA	12.17 (0.38)	10.91 (0.64)	2.652	0.944	0.082	3.069	NS	NS
Moderate EPA	12.32 (0.50)	11.88 (0.70)	0.075	0.334	0.921	0.083		
High EPA	13.00 (0.33)	13.06 (0.42)	0.05	0.009	0.002	0.03		
**T5 score**								
Low EPA	12.57 (0.38)	12.55 (0.51)	0.41	0.103	0.059	2.101	NS	NS
Moderate EPA	12.32 (0.50)	12.18 (0.58)	0.665	0.749	0.942	0.15		
High EPA	12.50 (0.41)	12.75 (0.37)	0.008	0.001	0.001	0.021		
**VLMT interference parameters**	EPA: *F*(6,198) = 1.426, *p* = 0.206, η_p_^2^ = 0.041 S: *F*(3,98) = 1.056, *p* = 0.372, η_p_^2^ = 0.031 I: *F*(6,198) = 0.102, *p* = 0.996, η_p_^2^ = 0.003 G: *F*(3,98) = 0.869, *p* = 0.460, η_p_^2^ = 0.026							
**I**								
Low EPA	6.43 (0.40)	6.18 (0.62)	1.671	2.28	0.079	1.414	NS	NS
Moderate EPA	6.41 (0.44)	5.71 (0.58)	0.193	0.134	0.924	0.237		
High EPA	7.33 (0.71)	6.63 (0.52)	0.032	0.022	0.002	0.014		
**T6**								
Low EPA	11.96 (0.59)	11.55 (0.56)	2.364	0.68	0.134	1.917	NS	NS
Moderate EPA	11.36 (0.56)	10.94 (0.70)	0.101	0.411	0.875	0.169		
High EPA	12.28 (0.43)	12.44 (0.48)	0.045	0.007	0.003	0.019		
**T5–6**								
Low EPA	0.61 (0.43)	1.00 (0.54)	2.556	0.756	0.075	0.107	NS	NS
Moderate EPA	0.95 (0.24)	1.24 (0.36)	0.083	0.387	0.928	0.744		
High EPA	0.22 (0.31)	0.31 (0.37)	0.049	0.008	0.001	0.001		
**VLMT long-term memory parameters**	EPA: *F*(4,200) = 0.604, *p* = 0.660, η_p_^2^ = 0.012 S: *F*(2,99) = 0.055, *p* = 0.946, η_p_^2^ = 0.001 I: *F*(4,200) = 0.093, *p* = 0.984, η_p_^2^ = 0.002 G: *F*(2,99) = 1.123, *p* = 0.330, η_p_^2^ = 0.022							
**T7**								
Low EPA	11.78 (0.54)	11.82 (0.48)	1.1	0.091	0.157	1.832	NS	NS
Moderate EPA	11.55 (0.67)	11.18 (0.69)	0.337	0.763	0.855	0.179		
High EPA	12.00 (0.46)	12.44 (0.58)	0.022	0.001	0.003	0.001		
**T5–7**								
Low EPA	0.78 (0.36)	0.73 (0.41)	0.774	0.007	0.123	0.128	NS	NS
Moderate EPA	0.77 (0.44)	1.00 (0.39)	0.464	0.934	0.884	0.721		
High EPA	0.50 (0.31)	0.31 (0.44)	0.015	0	0.002	0.001		
	Moderate depression *N* = 62 *M (SD)*	Severe depression *N* = 44 *M (SD)*						
**Digits forward**								
Low EPA	8.68 (0.44)	8.64 (0.41)	0.654	0.018	0.019	0.043	NS	NS
Moderate EPA	8.45 (0.37)	8.29 (0.49)	0.522	0.894	0.981	0.837		
High EPA	8.89 (0.40)	8.88 (0.52)	0.013	0	0	0		
**Digits backward**								
Low EPA	8.09 (0.41)	7.64 (0.49)	0.556	1.016	0.188	0.151	NS	NS
Moderate EPA	8.41 (0.47)	7.88 (0.36)	0.575	0.316	0.829	0.699		
High EPA	8.33 (0.43)	8.31 (0.27)	0.011	0.01	0.004	0.002		

Note: η_p_^2^ = 0.01 (small), 0.06 (medium), and 0.14 (large). l, low EPA; m, moderate EPA; h, high EPA. Group sizes (*n*): Moderately depressed with a low EPA status = 23; moderately depressed with a moderate EPA status = 22; moderately depressed with a high EPA status = 18; severely depressed with a low EPA status = 11; severely depressed with a moderate EPA status = 17; severely depressed with a high EPA status = 16. Multivariate effects, pairwise comparisons: ° borderline significant, * *p* < 0.05 ** *p* < 0.01. Between-subjects effects: ° borderline significant, * *p* < 0.010 (short-term memory), * *p* < 0.017 (interference), and * *p* < 0.025 (long-term memory) after Bonferroni correction of the significance level. Significant effects are marked in bold. T1–7, trials 1–7; NS, not significant.

**Table 4:** MANCOVA and ANCOVA results for the VLMT and digit span parameters.

	MANCOVA DHA Status (DHA) Depression Severity (S) Interaction Severity * DHA Status (I) Covariates: Gender (G), GIX (IQ)								
VLMT short-term memory parameters	DHA: *F*(10,178) = 1.323, *p* = 0.221, η_p_^2^ = 0.069 S: *F*(5,88) = 0.524, *p* = 0.758, η_p_^2^ = 0.029 I: *F*(10,178) = 0.686, *p* = 0.736, η_p_^2^ = 0.037 G: *F*(5,88) = 1.218, *p* = 0.308, η_p_^2^ = 0.065 IQ: *F*(5,88) = 6.113, *p* < 0.001 ***, η_p_^2^ = 0.258								
	Moderate depression *N* = 59 *M (SD)*	Severe depression *N* = 41 *M (SD)*	*F* *p* ηp^2^					Pairwise	*p*
			DHA	S	I	G	IQ		
**T1 score**									
Low DHA	6.65 (1.61)	6.40 (2.07)	1.57	0.057	0.115	0.073	**7.401**	NS	NS
Moderate DHA	7.25 (1.68)	7.00 (1.41)	0.214	0.812	0.892	0.788	0.008 *		
High DHA	6.94 (1.61)	6.78 (1.90)	0.033	0.001	0.002	0.001	**0.074**		
**T2 score**									
Low DHA	9.87 (2.97)	8.91 (0.69)	1.733	0.923	0.323	0	**14.652**	NS	NS
Moderate DHA	10.85 (2.87)	9.10 (2.13)	0.182	0.339	0.725	0.982	<0.001 *		
High DHA	10.25 (1.18)	10.28 (2.63)	0.036	0.01	0.007	0	**0.137**		
**T3 score**									
Low DHA	11.09 (2.17)	11.50 (2.72)	1.096	0.184	0.641	3.039	**15.105**	NS	NS
Moderate DHA	11.70 (2.06)	10.38 (2.06)	0.338	0.669	0.529	0.085	<0.001 *		
High DHA	11.75 (2.05)	11.94 (1.86)	0.023	0.002	0.014	0.032	**0.141**		
**T4 score**									
Low DHA	11.96 (2.35)	12.20 (2.20)	1.769	0.1	0.913	2.062	**31.252**	NS	NS
Moderate DHA	13.15 (1.73)	11.54 (3.05)	0.176	0.752	0.405	0.154	<0.001 *		
High DHA	12.63 (1.09)	13.06 (1.51)	0.037	0.001	0.019	0.022	**0.254**		
**T5 score**									
Low DHA	12.09 (2.28)	13.10 (1.60)	0.038	0.366	1.816	0.881	**19.51**	NS	NS
Moderate DHA	13.05 (1.79)	11.77 (1.88)	0.963	0.547	0.169	0.35	<0.001 *		
High DHA	12.44 (1.63)	12.89 (1.64)	0.001	0.004	0.038	0.009	**0.175**		
**VLMT interference parameters**	DHA: *F*(6,182) = 0.933, *p* = 0.472, η_p_^2^ = 0.039 S: *F*(3,90) = 0.716, *p* = 0.545, η_p_^2^ = 0.023 **I: *F*(6,182) = 2.219, *p* = 0.052 °, η_p_^2^ = 0.066** G: *F*(3,90) = 0.480, *p* = 0.697, η_p_^2^ = 0.016 IQ: *F*(3,90) = 8.593, *p* = < 0.001 ***, η_p_^2^ = 0.223								
**I**									
Low DHA	7.13 (2.70)	5.90 (2.38)	0.066	0.804	0.753	1.131	**16.398**	NS	NS
Moderate DHA	6.60 (1.76)	5.92 (2.63)	0.936	0.372	0.474	0.29	<0.001 *		
High DHA	6.25 (2.46)	6.39 (1.91)	0.001	0.009	0.016	0.012	**0.151**		
**T6**									
Low DHA	11.43 (2.76)	12.70 (2.36)	1.405	0.006	**4.337**	0.445	**12.435**	I: s/h > s/m I: m/m > s/m	*p* = 0.036 * *p* = 0.021 *
Moderate DHA	12.45 (2.21)	10.00 (2.35)	0.251	0.936	0.016 *	0.506	0.001 *		
High DHA	12.06 (1.95)	12.61 (1.29)	0.03	0	**0.086**	0.005	**0.119**		
**T5–6**									
Low DHA	0.65 (1.56)	0.40 (1.58)	2.621	0.613	1.959	0.017	0.006	NS	NS
Moderate DHA	0.60 (1.60)	1.77 (1.64)	0.078	0.436	0.147	0.897	0.936		
High DHA	0.38 (1.41)	0.28 (1.32)	0.054	0.007	0.041	0	0		
**VLMT Long-term memory parameters**	DHA: *F*(4,184) = 0.599, *p* = 0.664, η_p_^2^ = 0.013 S: *F*(2,91) = 0.181, *p* = 0.835, η_p_^2^ = 0.004 I: *F*(4,184) = 1.805, *p* = 0.130, η_p_^2^ = 0.038 G: *F*(2,91) = 0.488, *p* = 0.615, η_p_^2^ = 0.011 IQ: *F*(2,91) = 11.721, *p* < 0.001 ***, η_p_^2^ = 0.205								
**T7**									
Low DHA	11.70 (2.79)	11.90 (2.51)	0.804	0.151	1.598	0.744	**19.794**	NS	NS
Moderate DHA	12.25 (2.95)	10.62 (2.18)	0.45	0.698	0.208	0.391	<0.001 *		
High DHA	11.69 (2.02)	13.00 (1.33)	0.017	0.002	0.034	0.008	**0.177**		
**T5–7**									
Low DHA	0.39 (1.64)	1.20 (1.75)	1.11	0.011	1.86	0.031	1.802	NS	NS
Moderate DHA	0.80 (2.12)	1.15 (1.46)	0.334	0.916	0.161	0.86	0.183		
High DHA	0.75 (1.44)	−0.11 (1.41)	0.024	0	0.039	0	0.019		
	Moderate depression *N* = 58 *M (SD)*	Severe depression *N* = 41 *M (SD)*							
**Digits forward**									
Low DHA	9.09 (2.18)	9.90 (1.37)	**3.342**	0.956	1.523	0.23	**25.145**	NS	NS
Moderate DHA	8.45 (1.50)	7.85 (1.41)	0.040 *	0.543	0.224	0.633	<0.001 *		
High DHA	8.69 (1.66)	8.28 (2.08)	**0.068**	0.004	0.032	0.003	**0.216**		
**Digits backward**									
Low DHA	8.64 (2.36)	8.60 (1.17)	0.78	0.005	0.192	0.058	**17.851**	NS	NS
Moderate DHA	8.15 (1.81)	7.62 (1.26)	0.461	0.942	0.826	0.81	<0.001 *		
High DHA	8.19 (1.64)	7.89 (1.61)	0.017	0	0.004	0.001	**0.164**		

Note: GIX, global intelligence index. η_p_^2^ = 0.01 (small), 0.06 (medium), and 0.14 (large). L, low DHA status; m, moderate DHA status; h, high DHA status; m/l, moderately depressed with a low DHA status; m/m, moderately depressed with a moderate DHA status; m/h, moderately depressed with a high DHA status; s/l, severely depressed with a low DHA status; s/m, severely depressed with a moderate DHA status; s/h, severely depressed with a high DHA status. Group sizes (*n*): m/l = 23; m/m = 20; m/h = 16; s/l = 10; s/m = 13; s/h = 18. Multivariate effects, pairwise comparisons: ° borderline significant * *p* < 0.05, *** *p* < 0.001. Between-subjects effects: ° borderline significant, * *p* < 0.010 (short-term memory), * *p* < 0.017 (interference), and * *p* < 0.025 (long-term memory) after Bonferroni correction of the significance level. Significant effects are marked in bold. T1–7, trials 1–7; NS, not significant.
